# Acute pulmonary embolism with arrhythmia associated with minimal change disease in adults: a case report

**DOI:** 10.3389/fcvm.2023.1182569

**Published:** 2023-08-08

**Authors:** Chunyan Rong, Xuhan Liu, Baoguo Wang, Weihua Zhang

**Affiliations:** Department of Cardiovascular Medicine, The First Hospital of Jilin University, Changchun, China

**Keywords:** minimal change disease, pulmonary embolism, arrhythmia, MCD, VTE

## Abstract

**Background:**

Minimal change disease (MCD) is a common pathological type of nephrotic syndrome (NS), and is one of the most common causes of NS in children, but is not common in adults. MCD is sensitive to corticosteroid therapy and has a good prognosis, but is prone to relapse. Venous thromboembolism (VTE) is less common in MCD.

**Case presentation:**

We report a case of acute pulmonary embolism (PE) with arrhythmia associated with MCD in adults. The hypercoagulable state caused by MCD through multiple systems may be one of the important causes of thrombosis in this patient. In addition to the conventional corticosteroid therapy, he was started on anticoagulation for VTE and PE. His hospital course was complicated by atrial tachyarrhythmias initially controlled by amiodarone but he required readmission due to recurrent atrial flutter. His clinical condition became more stable after radiofrequency ablation.

**Conclusion:**

VTE associated with MCD in adults is rare. Treatment of MCD with corticosteroids may be associated with a higher risk of developing blood clots. This type of case is relatively rare and should be paid attention to. The mechanism of VTE in MCD is still a direction worthy of further research.

## Background

MCD is one of the five common pathologic subtypes of glomerular disease. MCD can be primary or secondary. Renal biopsy is the gold standard for the diagnosis of MCD. Thromboembolic disease is considered to be the most important complication of adult nephrotic syndrome. Membranous nephropathy is the main pathological type but MCD accounts for 15% of NS in adults. Corticosteroid is a standard therapy for MCD but 10%–30% of adults with MCD may not respond to the treatment. In these patients, immunosuppressive therapy such as cyclosporin is recommended (1, 2). Acute pulmonary embolism associated with MCD in adults is very rare (3). We report a 62-year-old adult male patient diagnosed with MCD who developed acute pulmonary embolism with arrhythmia after corticosteroid therapy.

## Case presentation

**Figure 1:**
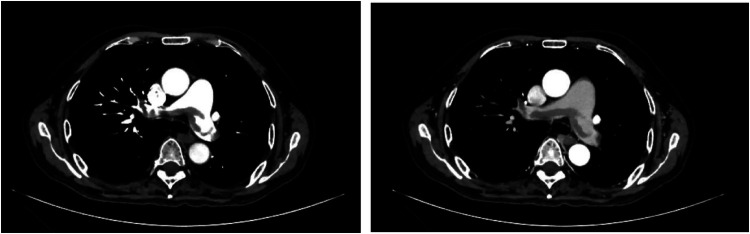
Both left and right images show multiple lamellar low-density filling defects in the pulmonary artery cavity of the main pulmonary artery, double pulmonary trunk, and multiple branches of each lobe of both lungs.

The 62-year-old male patient was transferred to our hospital due to a more than 3-week history of dyspnea and the recent onset of generalized fatigue. He was diagnosed with MCD 10 months prior (September 30, 2022) but had no history of other major medical issues. His vital signs were stable. Oxygen saturation was 95% on room air. Venous duplex ultrasound of the lower extremity showed acute deep venous thrombosis (DVT) in other hospitals. Echocardiography: slight enlargement in both chambers (Left atrial 38 mm, Right atrial 41 mm × 49 mm), severely increased pulmonary artery systolic pressure (85 mmHg). Based on the patient's clinical symptoms, signs, and auxiliary examination, the simplified Wells PE score was 2 points, and the patient was moderately likely to have a pulmonary embolism. D-dimer was extracted, and the patient was positive for D-dimer, and then computed tomography pulmonary angiography (CTPA) examination was performed, indicating pulmonary embolism in the main pulmonary artery, double pulmonary trunk, and multiple branches of each lobe of both lungs (Figure 1). Acute pulmonary embolism was eventually diagnosed.

In November 2021, he was diagnosed with MCD at the Second Hospital of Jilin University. At that time, albumin protein 37.0 g/L, Urine protein 2+, 24 h urine protein 2.24 g/24 h, the renal biopsy report showed that 36 intact glomeruli were observed in the whole film, 3 glomeruli had global sclerosis, 1 glomerular balloon dilated, and no adhesion or crescent formation was observed. Focal segmental hyperplasia of mesangial cells. Renal tubule epithelial cells showed multifocal granular and vacuolar degeneration, focal atrophy, and protein tubule type. Renal interstitial edema, small focal fibrosis, and inflammatory cell infiltration. Periodic acid-silver metheramine (PASM)+Masson staining showed no nail spike, double track, and erythrophilic material deposition. Pathological diagnosis: minor glomerular lesions. Electron microscope observation showed that mesangial cells and stroma of the glomerulus had slight segmental hyperplasia, basement membrane was segmental thickening, segmental shrinkage, no definite electron compact deposition, and epithelial foot process fusion of about 50%. Lysosomes of renal tubule epithelial cells increased, and part of the tubule microvilli shed, shedding cell fragments can be seen. Electron microscopic diagnosis: podocyte injury. Immunofluorescence was negative. Corticosteroid therapy was recommended and was considered by the patient and his family. Regular corticosteroid therapy was not initiated until July 2022, when the lack of remission persisted.

He was admitted to our department for acute pulmonary embolism on this occasion, and relevant laboratory tests were completed (Table 1): Echocardiography showed normal left ventricular systolic function, LVEF of 57%, right atrial and right ventricular enlargement, elevated pulmonary artery systolic pressure, and decreased right ventricular systolic function. Venous duplex ultrasound of the lower extremities showed thrombus in the left popliteal vein and posterior tibial vein (acute—subacute stage), and bilateral intermuscular vein thrombosis (acute—subacute stage). Upon admission, the patient had a blood pressure of 110/76 mmHg, a heart rate of 77 times/min, and his vital signs were stable; however, he had elevated BNP and RV dysfunction. His sPESI score was 0, therefore he was determined to be in the low to moderate risk in terms of his PE. He was subsequently started on enoxaparin sodium 4000IU parenteral anticoagulation every 12 h for 7 days followed by oral rivaroxaban 20 mg once a day. The patient developed atrial tachyarrhythmias during the initial part of his hospitalization. Electrocardiography (ECG) during tachycardia first indicated ectopic atrial rhythm and atrial flutter with 2:1 AV conduction. He then went into atrial fibrillation approximately 30 min later. He was given amiodarone which helped restored sinus rhythm. He remained in sinus rhythm during the rest of his hospital stay. Combined with the expert advice of the nephrology department, corticosteroid 32 mg once a day was used to treat the primary disease. After treatment, the patient was discharged from hospital with improved symptoms.

**Table 1:** Comparison of laboratory results between two admissions.

Inspection item	First admission result	Second admission result	Unit	Range of reference
Temperature	36.7	36.5	°C	36.0–37.0^a^
Blood pressure	110/76	97/60	mmHg	90/60–140/90
Heart rate	77	158	times/min	60–100
Respiratory frequency	18	18	times/min	12–20
blood oxygen saturation	95	93	%	93–98
D- dimer	4,920	894	ng/ml	100–600
BNP	2,070	1,370	pg/ml	0–100
PTA	138	104	%	80–120
Plasma antithrombin	108.2		%	80.0–130.0
Protein C activity	161		%	70–140
Protein S activity	142.7		%	63.5–149.0
CRP	21.62	3.28	mg/L	0–1.0
Urine protein	1+	+−		Negative
Urinary occult blood	+−	−		Negative
Albumin protein	28.3	23.9	g/L	40.0–55.0
TC	6.94	5.76	mmol/L	2.6–6.0
TG	2.42	2.99	mmol/L	0.28–1.80
LDL-C	4.7	3.95	mmol/L	<4.14^b^
Complement C3	1.78		g/L	0.7–1.4
Anti-β2-glycoprotein I antibody	25		RU/ml	0–20
24 h urine protein	0.714	0.504	g/24h	<0.15
24 h urine microalbumin	372.74	226.15	mg/24h	0–30
Fecal occult blood	+	+		Negative
HGB	111	141	g/L	130–175

BNP, brain natriuretic peptide; PTA, prothrombin activity; CRP, C-reactive protein; TC, total cholesterol; TG, triglyceride; LDL-C, low-density lipoprotein cholesterol; HGB, hemoglobin; The date of the first admission result was September 30, 2022. The date of the second admission result was November 2, 2022.

^a^ Measurement of axillary temperature.

^b^ Low risk—Target value <4.14; Medium dangerous-target value <3.37; High-risk—target

value <2.59; Very High risk—Target value <2.07.

**Figure 2:**
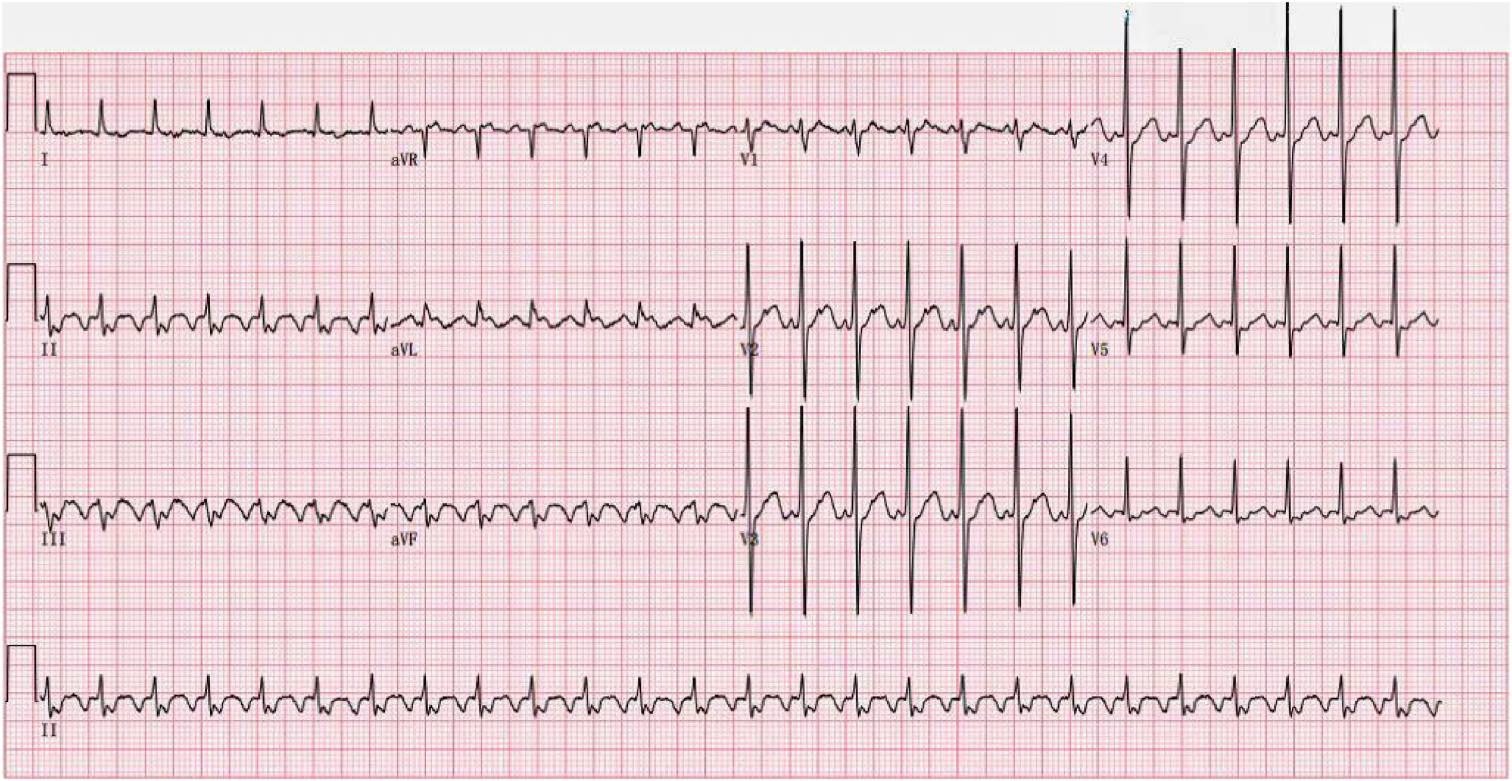
ECG of the patient after the second admission (heart rate 156 beats/min, ectopic rhythm, abnormal ECG, atrial flutter, 2:1 AV conduction).

**Figure 3:**
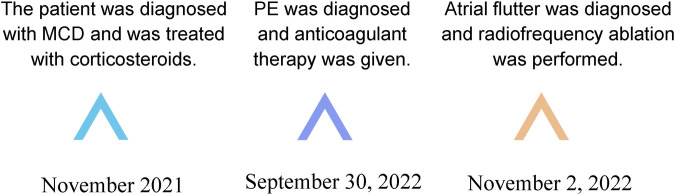
A timeline of all the patient's events.

One month later, he returned on November 2, 2022, due to an “increased heart rate for one day”. Admission ECG showed atrial flutter, 2:1 AV conduction (Figure 2); The patient's heart rate increased and remained unrelieved. The sinus rhythm was restored after “intravenous amiodarone bolus.” Echocardiography showed normal left ventricular systolic function, LVEF of 60%, enlargement of both atria, possible moderately increased pulmonary artery systolic pressure, and slightly reduced right heart function. The decision was made to perform radiofrequency ablation, considering that the rapid heart rate caused by atrial flutter could aggravate heart failure. After completing left atrial computed tomography angiography (CTA) and transesophageal echocardiography, no obvious abnormalities were found. Then bilateral annular pulmonary vein ablation and isthmic ablation of the tricuspid valve were performed, and sinus rhythm was restored after surgery (Figure 3). After discharge, Rivaroxaban tablets were given 20 mg once a day anticoagulant therapy, and oral corticosteroid 24 mg daily was continued to treat MCD. Patients were asked to follow up regularly.

## Discussion and conclusion

Minimal Change disease (MCD) is one of the five common pathological subtypes of glomerular disease, characterized by macroalbuminuria and hypoalbuminemia, resulting in edema and hypercholesterolemia. Most patients with MCD are primary with no obvious associated underlying disease or event. This patient does not have other triggers secondary to extraglomerular and co-occurring during glomerular processes, such as nonsteroidal anti-inflammatory drugs, so MCD considerations in this patient are primary (1). Under normal circumstances, the synapse on the podocyte can prevent protein leakage out of the body, while MCD will make the podocyte functional change, leading to synapse fusion, resulting in reduced filtration area, larger pore size, increased permeability of glomerular basement membrane, so that the blood protein leakage, resulting in proteinuria (4). The renal biopsy of this patient lacked obvious glomerular lesions under the light microscope, and there was fusion and destruction of foot processes of visceral epithelial cells under the electron microscope. The vast majority of MCDs occur in children, with only 15 percent or less of adult cases (1, 2). As in children, most cases of MCD in adults are sensitive to corticosteroid therapy. However, the effect is worse than that of children, and the onset of the effect is slow. In some patients, the effect of corticosteroid treatment is 3–4 months, and effective in approximately 75% of patients. In more than half of patients, proteinuria will relapse after remission, and more than one third of adults will relapse frequently and become corticosteroid-dependent. Oral immunosuppressive agents can be used for adult MCD with frequent recurrence or corticosteroid-dependent disease (5). The patient is currently being treated with corticosteroids and is in stable condition. The association of the nephrotic syndrome with an increased risk of venous thromboembolism is clear. The risk of membranous nephropathy is the highest.

Some studies have suggested that corticosteroid therapy may increase the risk of VTE (6, 7), it affects more than 4.6 per 1,000 persons each year, and the mechanism may be related to the increase of corticosteroids in the body. Corticosteroids can increase the levels of coagulation factors and fibrinogen by directly activating coagulation and inhibiting the dissolution of fibrinogen (8–10). Corticosteroid is the standard of care for patients with MCD. This patient was at a significantly increased risk for VTE due to her advanced age and long-term corticosteroid therapy.

Acute pulmonary embolism associated with MCD in adults is very rare (3). The patient was pathologically diagnosed as MCD 1 year ago, with clinical manifestations of NS such as hypoalbuminemia (albuminemia <30 g/L is hypoalbuminemia) (5) and edema, hyperlipidemia (Table 1). Acute pulmonary embolism later occurred because of the large amount of albumin lost from urine due to podocyte disease, resulting in a decrease in plasma albumin due to the liver's increased compensatory synthesis of proteins along with the synthesis of clotting factors. In addition, hyperlipidemia increased the blood viscosity and edema caused by low plasma colloid osmotic pressure reduced the blood volume, causing the imbalance of coagulation, anticoagulation, and fibrinolytic systems in the body, so that the blood was in a hypercoagulable state (11, 12). There are a variety of reasons why the patient's blood was in a hypercoagulable state, eventually leading to the occurrence of VTE. However, the cause of VTE is uncertain. Prolonged immobilization was not related, since the patient reported regular daily activity. The patient had a history of MCD, D-dimer 4,920 ng/ml, lower extremity venous color ultrasound suggested deep vein thrombosis, and CTPA suggested pulmonary embolism, which was considered to be hypercoagulability of blood caused by MCD.

Pulmonary embolism leads to increased right ventricular afterload, right atrial and right ventricular dilatation, and right heart failure. Right atrial remodeling predisposes atrial arrhythmias such as atrial flutter and atrial fibrillation, which can aggravate heart failure. For patients with atrial arrhythmia combined with heart failure, radiofrequency ablation should be performed as soon as possible to restore sinus rhythm and improve heart function (13). At the second admission, the patient's BNP 1,370 pg/ml, D-dimer 894 ng/ml, Albumin protein 23.9 g/L, echocardiographic showed enlargement of both atria, and slightly reduced right heart function. Venous duplex ultrasound of the lower extremity showed subacute DVT. CTPA showed that the area of PE was significantly reduced. For atrial arrhythmias, bilateral circumferential pulmonary vein ablation and isthmus ablation were performed to eliminate atrial fibrillation and atrial flutter. As the patient's pulmonary embolism is associated with nephrotic syndrome, the primary disease MCD should be actively treated to prevent VTE recurrence. According to the 2020 Evidence-Based Clinical Practice Guidelines for Nephrotic syndrome (5), corticosteroid therapy is the standard treatment for MCD. In this case, the patient received oral maintenance therapy with corticosteroid under the guidance of a nephrologist.

In conclusion, the common pathological type of adult VTE-related nephrotic syndrome is usually membranous nephropathy, and this case was caused by adult MCD, which is relatively rare in clinical practice.

## Data Availability

The original contributions presented in the study are included in the article/Supplementary Materials, further inquiries can be directed to the corresponding author.
